# Output Feedback Adaptive Tracking Control for Uncertain Strict-Feedback Nonlinear Systems with Full-State Constraints and Unknown Output Gain

**DOI:** 10.3390/s26103084

**Published:** 2026-05-13

**Authors:** Zhenlin Wang, Seiji Hashimoto, Pengqiang Nie, Song Xu, Takahiro Kawaguchi

**Affiliations:** 1 Division of Electronics and Informatics, Gunma University, Kiryu 376-8515, Japan; zhenlinwang8689@gmail.com (Z.W.);; 2 College of Automation, Jiangsu University of Science and Technology, Zhenjiang 212000, China

**Keywords:** nonlinear system, unknown output gain, full-state constraints, adaptive backstepping control, tracking control

## Abstract

In this paper, an adaptive output feedback control scheme is proposed for a class of parametric strict feedback systems with asymmetric full-state constraints and unknown output gain. Firstly, an adaptive state observer is constructed to estimate the unmeasured system states. To compensate for the effect of the unknown output gain on the tracking performance, a new error signal incorporating an adaptive compensation coefficient is introduced into the backstepping design. Then, by combining the universal transformed function with a coordinate transformation, all system states are kept within time-varying asymmetric bounds, and the feasibility issues of conventional constrained control methods are avoided. Based on Lyapunov stability analysis, all signals in the closed-loop system are proven to be globally uniformly ultimately bounded. Finally, simulation results based on motor models demonstrate the effectiveness of the proposed scheme.

## 1. Introduction

Recently, various modern industrial systems can be modeled as nonlinear dynamical systems, such as industrial manipulators [1], computer numerical control machine tools [2], electromechanical systems [3,4], and renewable energy systems [5]. In practice, these nonlinear systems are often affected by complex disturbances and performance degradation associated with unknown output gain [6,7]. In the presence of unknown output gain, it is essential to guarantee that all state variables remain within safe and performance-specified ranges throughout the entire operating period, thereby preventing instabilities [8], structural damage [9], or loss of control [10].

In nonlinear systems, output feedback control is important when full-state measurements are unavailable [11,12,13]. However, the output signal may be affected by unknown output gain during operation, which may degrade the control performance [14]. Several studies have been conducted to address this issue. The authors of [15] proposed an output-feedback compensation control strategy that combines a switching adaptive observer with a backstepping-based controller to handle unknown output gain in nonlinear systems while guaranteeing bounded convergence and enhanced transient performance. Furthermore, the authors of [16] investigated output-feedback adaptive compensation for a class of parametric strict feedback systems with unknown output gain by employing a switching-type adaptive state observer and an adaptive compensation mechanism. However, this work mainly focused on compensating for unknown output gain and improving transient performance, without taking state constraints into account. In addition, the authors of [17] extended the related compensation framework to nonlinear, switched, interconnected systems under an average dwell time by developing a switched state observer and an adaptive compensation scheme. Nevertheless, the main objective of this work was still output tracking and boundedness analysis of closed-loop signals, and the issue of asymmetric time-varying full-state constraints was not addressed. Motivated by the above observations, it is still necessary to investigate how to simultaneously achieve unmeasured-state reconstruction, unknown output gain compensation, and asymmetric time-varying full-state constraint satisfaction for uncertain strict feedback nonlinear systems under an output-feedback framework.

In practical systems, especially in mechanical and electromechanical applications, state variables are often subject to physical and safety constraints. These constraints are usually asymmetric due to practical operational requirements [18,19]. Violating these constraints may cause system performance deterioration or even catastrophic consequences [20]. Therefore, constraint satisfaction must be considered in controller design [21]. As an effective approach for enforcing such constraints, the barrier Lyapunov function (BLF) method has been extensively utilized to ensure that state constraints are rigorously satisfied. For instance, the authors of [22] proposed a distributed fuzzy optimal consensus controller using a BLF-based control scheme, while the authors of [23] developed a neuro-adaptive optimized controller via the same method to handle state constraints. To enhance the convergence performance while avoiding the explosion of complexity problem, in [24,25], BLF-based control was integrated with dynamic surface control and finite-time and fixed-time control strategies. The authors of [26] solved the state constraint problem by introducing the universal transformed function and coordinate transformation techniques. However, the corresponding controller design depends on accurate measurement information. Once the output signal is affected by unknown output gain, the measured information may become unreliable, which may further weaken the constraint-handling mechanism that relies on accurate state information, making it difficult to guarantee constraint satisfaction.

From the above discussions on unknown output gain compensation and state constraint control, it can be observed that most existing studies have addressed these two issues separately, while their simultaneous consideration has received limited attention. On the one hand, many existing results have developed observers and adaptive compensation mechanisms for unknown output gain [15,16,17], while state constraints are rarely considered in these works. On the other hand, existing constraint-handling methods can effectively guarantee state confinement when accurate output information is available [22,23,24,25,26], but they usually rely on accurate measurement information. Therefore, for uncertain strict feedback nonlinear systems, it remains challenging to simultaneously achieve unmeasured-state reconstruction, unknown output gain compensation, and asymmetric time-varying full-state constraint satisfaction under an output-feedback framework.

Motivated by the preceding discussions, an adaptive output-feedback tracking control approach is developed for nonlinear systems subject to asymmetric constraints and unknown output gain. Based on the abovementioned discussions, the main contributions are summarized as follows:

(1) An adaptive state observer is developed to reconstruct the unmeasured states under unknown output gain. By introducing a new error signal with an adaptive compensation coefficient into the observer-based backstepping design, together with the corresponding adaptive updating laws, the adverse effect of unknown output gain on tracking performance can be effectively reduced.
(2) To handle asymmetric time-varying full-state constraints, a control design combining the universal transformed function with a novel coordinate transformation is proposed. The developed method guarantees that all system states remain within the prescribed asymmetric constraint boundaries throughout the entire operation process, without requiring the strict feasibility conditions encountered in conventional BLF-based approaches.
(3) By integrating adaptive state estimation and constraint-handling mechanisms into a single design, the proposed approach addresses the challenge of guaranteeing constraint satisfaction when the available output information is affected by unknown gain. Different from the compensation-based control methods in [16,17], where asymmetric full-state constraints are not explicitly addressed, the proposed method further incorporates a constraint-handling mechanism into the output-feedback control design. In addition, compared with the constrained control methods in [26], where adaptive compensation for unknown output gain was not considered, an adaptive compensation mechanism is incorporated in the proposed design to effectively reduce the influence of unknown output gain while ensuring constraint satisfaction.

This paper is organized as follows. Section 2 introduces the necessary background knowledge and presents the problem formulation. Section 3 presents a universal transformed function, the adaptive state observer design, the backstepping-based tracking controller design, and the stability analysis. The effectiveness of the proposed control scheme is verified by numerical simulations in Section 4. Finally, Section 5 concludes this paper.

## 2. Problem Statement and Preliminaries

### 2.1. Problem Statement

In this paper, the parameter strict feedback system is considered as follows:

(1) $$\left\{\begin{array}{c} {\dot{x}}_{1}=x_{2}+\theta^{T}\varphi_{1}\left(x_{1}\right)+d_{1}\left(t\right)\\ {\dot{x}}_{i}=x_{i+1}+\theta^{T}\varphi_{i}\left(x_{1},{\bar{x}}_{i}\right)+d_{i}\left(t\right)\\ {\dot{x}}_{n}=u+\theta^{T}\varphi_{n}\left(x_{1},{\bar{x}}_{n}\right)+d_{n}\left(t\right)\\ y=x_{1} \end{array}\right.$$

where i=2,…,n−1 and ${\bar{x}}_{i}=\left[x_{2},\ldots ,x_{i}\right]$. Here, $x={\left[x_{1},{\bar{x}}_{n}^{T}\right]}^{T}$ is the state vector, and u∈R represents the input of the system, while y∈R denotes the measured output, $\theta \in R^{p}$ represents the unknown parameter vector, and $\varphi_{1}\left(x_{1}\right)\in R^{p}$ and $\varphi_{1}\left(x_{1},{\bar{x}}_{i}\right)\in R^{p}$ are defined as known smooth nonlinear vector functions for i=2,…,n. Moreover, $d_{i}\left({\bar{x}}_{n}\right)\in R$ for i=1,…,n is defined as a state-dependent unknown uncertainty.

**Remark** **1.** *The system in Equation* (1) *has been widely studied in the literature [27,28]. It is widely employed to model various practical nonlinear systems, including motor systems, chemical reactors, and robotic systems.*

In practical engineering systems, the available output signal may be affected by environmental disturbances, hardware degradation, calibration errors, or data acquisition uncertainties. These factors can be lumped into an unknown output gain, which directly scales the output signal and may significantly influence the control performance.

**Definition** **1.** *The output signal W(t)∈R is affected by an unknown output gain after time $T_{g}$ if the available output signal satisfies*

(2) $$W^{F}\left(t\right)=\rho W\left(t\right),\;\forall t>T_{f},\;0<\rho <1$$

Based on Definition 1, the unknown output gain of the system in Equation (1) can be described as follows:

(3) $$y^{F}\left(t\right)=\rho y\left(t\right),\;0<\rho <1$$

**Remark** **2.** *It should be noted that the multiplicative factor ρ(t) represents a lumped gain degradation in the output channel. Physically, such attenuation could stem from several sources, including but not limited to sensor sensitivity loss, mechanical coupling degradation, or plant efficiency decrease. Since this paper does not employ an isolation mechanism, we treat ρ(t) as a general output gain uncertainty. The focus is on ensuring tracking performance under such degradation rather than identifying its specific physical origin.*

Define $\chi_{1}=\rho x_{1}$ and $\kappa =\frac{1}{\rho }$. Then, the system model is equivalent to

(4) $$\left\{\begin{array}{c} {\dot{\chi }}_{1}=\rho x_{2}+\theta_{1}^{T}\phi_{1}\left(\kappa \chi_{1}\right)+\rho d_{1}\left(t\right)\\ {\dot{x}}_{i}=x_{i+1}+\theta_{i}^{T}\phi_{i}\left(\kappa \chi_{1},{\bar{x}}_{i}\right)+d_{i}\left(t\right)\\ {\dot{x}}_{n}=u+\theta_{n}^{T}\phi_{n}\left(\kappa \chi_{1},{\bar{x}}_{n}\right)+d_{n}\left(t\right)\\ y^{F}=\chi_{1} \end{array}\right.$$

where $\theta_{1}=\rho \theta$, $\theta_{i}=\theta$, i=2,…,n, and ${\hat{\theta }}_{f}={\left[{\hat{\theta }}_{1},\ldots ,{\hat{\theta }}_{p}\right]}^{T}$. Nevertheless, in the controller design, $\chi_{1}$ is available.

The control objective is to develop an adaptive observer-based output-feedback tracking control scheme such that the following are true:

(1) The closed-loop signals in the system are globally uniformly ultimately bounded.
(2) The system output *y* is required to follow the reference trajectory $y_{d}\left(t\right)$ while ensuring that all system states remain within the time-varying asymmetric boundaries:
  (5) $$\begin{array}{c} \Omega_{\chi_{1}}=\left\{\chi_{1}\;|\;{\bar{H}}_{1}\left(t\right)>\chi_{1}\left(t\right)-y_{d}\left(t\right)>-{\underline{H}}_{1}\left(t\right)\right\} \end{array}$$
  (6) $$\begin{array}{c} \Omega_{x_{i}}=\left\{x_{i}\;|\;{\bar{H}}_{i}\left(t\right)>x_{i}\left(t\right)>-{\underline{H}}_{i}\left(t\right),\;i=2,\ldots ,n\right\} \end{array}$$
  In this formulation, ${\underline{H}}_{i}\left(t\right)$ and ${\bar{H}}_{i}\left(t\right)$ are positive and twice differentiable constraint functions, respectively.

**Remark** **3.** *For the time-varying asymmetric boundaries, the tracking error $y-y_{d}$ and states $x_{i}$ remain in the constraint boundaries throughout the operation. Specifically, if ${\underline{H}}_{i}\left(t\right)={\bar{H}}_{i}\left(t\right)$, then the constraint boundaries become symmetric.*

### 2.2. Preliminaries

To accomplish the above control objective, several useful assumptions are necessary to present, and they are as follows.

**Assumption** **1.** *Considering the unknown output gain model in Equation (3), the gain coefficient ρ satisfies $\rho \geq \underline{\rho }$, where $\underline{\rho }$ is a known positive constant.*

**Assumption** **2.** *The unknown parameter vector θ is bounded, and it satisfies $∥\theta ∥\leq \theta_{M}$, where $\theta_{M}$ is a positive constant.*

**Assumption** **3.** *The disturbance $d_{i}\left({\bar{x}}_{i}\right)$ is assumed to be bounded, and there is an unknown positive constant ${\bar{d}}_{i}$ such that $|d_{i}\left({\bar{x}}_{i}\right)|<{\bar{d}}_{i}$.*

**Assumption** **4.** *The reference signal $y_{d}$ and its derivatives ${\dot{y}}_{d}$, $y_{d}^{\left(2\right)},\ldots ,y_{d}^{\left(n\right)}$ are bounded.*

**Lemma** **1.** *For any continuous function F(a,b), where $a\in R^{M}$ and $b\in R^{N}$, there exist smooth functions X(a)≥1 and Y(b)≥1 such that |F(a,b)|≤X(a)Y(b).*

## 3. Output-Feedback Adaptive Tracking Control Design

### 3.1. Universal Transformed Functions

The tracking error is defined as $z_{1}=\hat{\kappa }\chi_{1}-y_{d}$, where $\hat{\kappa }$ is the adaptive compensation coefficient and will be determined later. Based on the constraint $\chi_{1}\in \Omega_{\chi_{1}}$ in Equation (5), it follows that $z_{1}\in \Omega_{z_{1}}:=\left\{z_{1}|-{\underline{H}}_{1}<z_{1}<{\bar{H}}_{1}\right\}$. To guarantee that $z_{1}$ remains within the prescribed constraint, the universal transformed function method is introduced as follows:

(7) $$\begin{array}{c} \varphi_{1}=\frac{{\underline{H}}_{1}{\bar{H}}_{1}z_{1}}{\left({\underline{H}}_{1}+z_{1}\right)\left({\bar{H}}_{1}-z_{1}\right)}=\frac{z_{1}}{\lambda_{1}} \end{array}$$

with the initial state $z_{1}\left(0\right)\in \left(-{\underline{H}}_{1},{\bar{H}}_{1}\right)$. From Equation (7), it follows that $\varphi_{1}\left(t\right)\to \infty$ as $z_{1}$ approaches its boundary. Specifically, for any initial condition $z_{1}\left(0\right)\in \left(-{\underline{H}}_{1},{\bar{H}}_{1}\right)$, we have $\varphi_{1}\left(t\right)\to \infty$ as $z_{1}\to {\bar{H}}_{1}$ or $z_{1}\to -{\underline{H}}_{1}$.

Based on the property of $\varphi_{1}\left(t\right)$, it can be concluded that for any $z_{1}\left(0\right)\in \Omega_{z_{1}}$, if $\varphi_{1}\left(t\right)$ is always bounded, then $z_{1}\left(t\right)\in \Omega_{z_{1}}$ holds for ∀t>0. By substituting the system model with unknown output gain in Equation (4) into ${\dot{\varphi }}_{1}$, it can be found that

(8) $$\begin{array}{c} {\dot{\varphi }}_{1}=\mu_{1}\left[\rho x_{2}+\theta_{1}\phi_{1}+\rho d_{1}-{\dot{y}}_{d}\right)]+\xi_{1} \end{array}$$

with

(9) $$\begin{array}{cc} \mu_{1} & =\frac{{\underline{H}}_{1}{\bar{H}}_{1}\left({\underline{H}}_{1}{\bar{H}}_{1}+z_{1}^{2}\right)}{{\left({\underline{H}}_{1}+z_{1}\right)}^{2}{\left({\bar{H}}_{1}-z_{1}\right)}^{2}} \end{array}$$

(10) $$\begin{array}{cc} \zeta_{1} & =\frac{-{\bar{H}}_{1}z_{1}^{2}{\dot{\underline{H}}}_{1}}{{\left({\underline{H}}_{1}+z_{1}\right)}^{2}\left({\bar{H}}_{1}-z_{1}\right)}-\frac{{\underline{H}}_{1}z_{1}^{2}{\dot{\bar{H}}}_{1}}{\left({\underline{H}}_{1}+z_{1}\right){\left({\bar{H}}_{1}-z_{1}\right)}^{2}}. \end{array}$$

Furthermore, to cope with the constraints of $x_{j}\;\left(j=2,\ldots ,n\right)$, the universal transformed functions are introduced as

(11) $$\begin{array}{c} \varphi_{j}=\frac{{\underline{H}}_{j}{\bar{H}}_{j}z_{j}}{\left({\underline{H}}_{j}+z_{j}\right)\left({\bar{H}}_{j}-z_{j}\right)}=\frac{z_{j}}{\lambda_{j}}. \end{array}$$

Based on the system in Equation (4) and the constraints in Equation (6), the dynamics of transformed states can be expressed as follows:

(12) $$\begin{array}{cc} \; & {\dot{\varphi }}_{j}=\mu_{j}\left[\lambda_{j+1}\varphi_{j+1}+\theta_{j}^{T}\phi_{j}+d_{j}\right]+\xi_{j},j=2,\ldots ,n-1 \end{array}$$

(13) $$\begin{array}{cc} \; & {\dot{\varphi }}_{n}=\mu_{n}\left[u+\theta_{n}^{T}\phi_{n}+d_{n}\right]+\xi_{n} \end{array}$$

where

(14) $$\begin{array}{cc} \mu_{j} & =\frac{{\underline{H}}_{j}{\bar{H}}_{j}\left({\underline{H}}_{j}{\bar{H}}_{j}+z_{j}^{2}\right)}{{\left({\underline{H}}_{j}+z_{j}\right)}^{2}{\left({\bar{H}}_{j}-z_{j}\right)}^{2}},\;\left(j=2,\ldots ,n\right) \end{array}$$

(15) $$\begin{array}{cc} \zeta_{j} & =\frac{-{\bar{H}}_{j}z_{j}^{2}{\dot{\underline{H}}}_{j}}{{\left({\underline{H}}_{j}+z_{j}\right)}^{2}\left({\bar{H}}_{j}-z_{j}\right)}-\frac{{\underline{H}}_{j}z_{j}^{2}{\dot{\bar{H}}}_{j}}{\left({\underline{H}}_{j}+x_{j}\right){\left({\bar{H}}_{j}-z_{j}\right)}^{2}}. \end{array}$$

### 3.2. Design of Adaptive State Observer

In the system in Equation (4), it is worth noting that the state variables $x_{2},\ldots ,x_{n}$ are unmeasurable in the control design, and a state observer should be constructed to estimate these states. Based on the designed observer, an adaptive output-feedback control strategy with an adaptive compensation mechanism for unknown output gain is proposed. The adaptive state observer is designed as follows:

(16) $$\left\{\begin{array}{c} {\dot{\hat{\chi }}}_{1}=\hat{\rho }{\hat{x}}_{2}+{\hat{\theta }}_{1}^{T}\phi_{1}\left({\hat{\kappa }}_{1}\chi_{1}\right)+k_{1}\left(y^{F}-{\hat{x}}_{1}\right)\\ {\dot{\hat{x}}}_{i}={\hat{x}}_{i+1}+{\hat{\theta }}_{i}^{T}\phi_{i}\left({\hat{\kappa }}_{1}\chi_{1},{\bar{\hat{x}}}_{i}\right)+k_{i}\left(y^{F}-{\hat{x}}_{1}\right)\\ {\dot{\hat{x}}}_{n}=u+{\hat{\theta }}_{n}^{T}\phi_{n}\left({\hat{\kappa }}_{1}\chi_{1},{\bar{\hat{x}}}_{n}\right)+k_{n}\left(y^{F}-{\hat{x}}_{1}\right) \end{array}\right.$$

where 2≤i≤n−1 and ${\hat{x}}_{1}$ denote the estimate of $\chi_{1}$ and ${\bar{\hat{x}}}_{i}={\left[{\hat{x}}_{2},\ldots ,{\hat{x}}_{i}\right]}^{T}$

Define the following observer error as $e={\left[e_{1},\ldots ,e_{n}\right]}^{T}$:

(17) $$\left\{\begin{array}{c} e_{1}=\chi_{1}-{\hat{x}}_{1},\\ e_{i}=x_{i}-{\hat{x}}_{i},\\ e_{n}=x_{n}-{\hat{x}}_{n}. \end{array}\right.$$

From Equations (4) and (16), the error equation of the state observer can be expressed as follows:

(18) $$\dot{e}=Ae+B_{1}\tilde{\rho }{\hat{x}}_{2}+F^{T}\left(\hat{\kappa }\chi_{1},{\hat{x}}_{n}\right)\tilde{\Theta }+\Delta F^{T}\Theta +B_{n}d$$

where $F\left(\hat{\kappa }\chi_{1},\hat{x}\right)=diag\left\{\phi_{1}\left({\hat{\kappa }}_{1}\chi_{1}\right),\ldots ,\phi_{n}\left({\hat{\kappa }}_{1}\chi_{1},{\hat{x}}_{n}\right)\right\}$, $\tilde{\rho }=\rho -\hat{\rho }$, ${\tilde{\theta }}_{i}=\theta_{i}-{\hat{\theta }}_{i}$, $\Delta F=diag\{\phi_{1}\left(\kappa_{1}\chi_{1}\right)-\phi_{1}\left({\hat{\kappa }}_{1}\chi_{1}\right),\ldots ,\phi_{n}\left(\kappa_{1}\chi_{1},{\bar{x}}_{n}\right)-\phi_{n}\left({\hat{\kappa }}_{1}\chi_{1},{\bar{\hat{x}}}_{n}\right)\}$, G=diag{ρ,1,…,1}, $B_{1}={\left[1\;0\;\cdots \;0\right]}^{T}$, $B_{n}={\left[0\;0\;\cdots \;1\right]}^{T}$, and

$$A=\left[\begin{array}{ccccc} -k_{1} & \rho & 0 & \cdots & 0\\ -k_{2} & 0 & 1 & \cdots & 0\\ \vdots & \vdots & \vdots & \; & \vdots \\ -k_{i} & \cdots & \cdots & 1 & 0\\ \vdots & \cdots & \cdots & 0 & 1\\ -k_{n} & \cdots & \cdots & 0 & 0 \end{array}\right],\;\tilde{\Theta }=\left[\begin{array}{c} {\tilde{\theta }}_{1}\\ \vdots \\ {\tilde{\theta }}_{n} \end{array}\right],\;\Theta =\left[\begin{array}{c} \theta_{1}\\ \vdots \\ \theta_{n} \end{array}\right]$$

Assume that there exists a positive definite matrix *P* such that

(19) $$A^{T}P+PA<-Q$$

where *Q* is symmetric positive definite.

Choose the Lyapunov candidate function $V_{0}$ to be

(20) $$V_{0}=e^{T}Pe+\frac{1}{2\gamma_{0}}{\tilde{\rho_{0}}}^{2}+\frac{M}{3\gamma_{1}}{\left|\tilde{\kappa }\right|}^{3}+\frac{1}{2\gamma_{2}}{\tilde{\Theta }}^{T}\tilde{\Theta }$$

where $M=\theta_{M}^{2}\sum_{i=1}^{n}L_{i}^{2}$ and $\tilde{\kappa }=\kappa -\hat{\kappa }$.

According to Equations (16) and (18), the time derivative of $V_{0}$ can be derived as follows:

(21) $$\begin{array}{cc} {\dot{V}}_{0}= & -e^{T}Qe+2e^{T}P\left[B_{1}\tilde{\rho }{\hat{x}}_{2}+F^{T}\left({\hat{\kappa }}_{1}\chi_{1},{\hat{x}}_{n}\right)\tilde{\Theta }+\Theta^{T}\Delta F+B_{n}d\right]\\ \; & -\frac{1}{\gamma_{0}}{\tilde{\rho }}_{0}{\dot{\hat{\rho }}}_{0}-\frac{M}{\gamma_{1}}{\tilde{\kappa }}^{2}\dot{\hat{\kappa }}\;\mathrm{sgn}\left(\tilde{\kappa }\right)-\frac{1}{\gamma_{2}}{\tilde{\Theta }}^{T}\dot{\hat{\Theta }} \end{array}$$

According to Assumptions 2 and 3 and Young’s inequality, the following inequalities can be obtained:

(22) $$\begin{array}{cc} \; & 2e^{T}PB_{1}\tilde{\rho_{0}}{\hat{x}}_{2}\leq 2e_{10}^{T}PB_{1}\tilde{\rho_{0}}{\hat{x}}_{2}+e^{T}{\bar{I}}_{1}PP{\bar{I}}_{1}e{\hat{x}}_{2}^{2}+{\tilde{\rho_{0}}}^{2} \end{array}$$

(23) $$\begin{array}{cc} \; & 2e^{T}PF^{T}\left({\hat{\kappa }}_{1}\chi_{1},{\bar{\hat{x}}}_{n}\right)\tilde{\Theta }\leq 2e_{10}^{T}PF^{T}\left({\hat{\kappa }}_{1}\chi_{1},{\bar{\hat{x}}}_{n}\right)\tilde{\Theta }+e^{T}{\bar{I}}_{1}PF^{T}\left({\hat{\kappa }}_{1}\chi_{1},{\bar{\hat{x}}}_{n}\right)F\left({\hat{\kappa }}_{1}\chi_{1},{\bar{\hat{x}}}_{n}\right)P{\bar{I}}_{1}e+{\tilde{\Theta }}^{T}\tilde{\Theta } \end{array}$$

(24) $$\begin{array}{cc} \; & 2e^{T}P\Delta F^{T}\Theta \leq e^{T}PPe+\theta_{M}^{2}\left[\sum_{i=1}^{n}L_{i}^{2}{\tilde{\kappa }}^{2}\chi_{1}^{2}+\sum_{i=2}^{n}L_{i}^{2}\sum_{j=2}^{n}e_{j}^{2}\right] \end{array}$$

(25) $$\begin{array}{cc} \; & 2e^{T}PB_{n}d\leq e^{T}PB_{n}B_{n}^{T}Pe+d_{M}^{2} \end{array}$$

where $e_{10}={\left[e_{1},0,\ldots ,0\right]}^{T}$, ${\bar{I}}_{1}=diag\left\{0,1,\ldots ,1\right\}\in R^{n\times n}$, and $e=\left[e_{1},0,\ldots ,0\right]+\left[0,e_{2},\ldots ,e_{n}\right]$.

Substituting Equations (22)–(25) into Equation (21) yields

(26) $$\begin{array}{cc} {\dot{V}}_{0}\leq & -e^{T}\bar{Q}e+\frac{1}{\gamma_{0}}{\tilde{\rho }}_{0}\left[2\gamma_{0}e_{10}^{T}PB_{1}{\hat{x}}_{2}-{\dot{\hat{\rho }}}_{0}\right]+\frac{M}{\gamma_{1}}{\tilde{\kappa }}^{2}\left[\gamma_{1}\chi_{1}^{2}-\dot{\hat{\kappa }}\;\mathrm{sgn}\left(\tilde{\kappa }\right)\right]\\ \; & +{\tilde{\rho }}_{0}^{2}+{\tilde{\Theta }}^{T}\tilde{\Theta }+\frac{1}{\gamma_{2}}{\tilde{\Theta }}^{T}\left[2\gamma_{2}F\left({\hat{\kappa }}_{1}\chi_{1},{\hat{x}}_{n}\right)Pe_{10}-\dot{\hat{\Theta }}\right]+d_{M}^{2} \end{array}$$

where $\bar{Q}=Q-PP-\left[{\hat{x}}_{2}^{2}+max_{2\leq i\leq n}\phi_{i}^{2}\left(\hat{\kappa }\chi_{1},{\bar{\hat{x}}}_{i}\right){\bar{I}}_{1}PP{\bar{I}}_{1}-\theta_{M}^{2}\sum_{i=2}^{n}L_{i}^{2}\bar{I_{1}}-PB_{n}B_{n}^{T}P\right]$.

The piecewise updating law for $\hat{\kappa }$ is designed as follows:

(27) $$\dot{\hat{\kappa }}={\mathrm{Proj}}_{\left[1,\;1/\varrho \right]}\left\{K\right\}=\left\{\begin{array}{cc} 0, & \mathrm{if}\;\hat{\kappa }=1\;\mathrm{and}\;K\leq 0\\ \; & \mathrm{or}\;\hat{\kappa }=1/\varrho \;\mathrm{and}\;K\geq 0\\ K, & \mathrm{otherwise} \end{array}\right.$$

where

(28) $$K=\left\{\begin{array}{cc} 0, & m\leq 0\\ \gamma_{1}\chi_{1}^{2}+\frac{\epsilon \;\gamma_{1}^{\prime }}{\epsilon \gamma_{1}^{\prime }+\chi_{1}^{2}}-\sigma_{1}\hat{\kappa }, & m>0 \end{array}\right.$$

where $m=\gamma_{1}\chi_{1}^{2}+\frac{\epsilon \;\gamma_{1}^{\prime }}{\epsilon \gamma_{1}^{\prime }+\chi_{1}^{2}}-\sigma_{1}\hat{\kappa }$. In the equation, Proj[·] denotes the projection operator, which ensures that the estimate $\hat{\kappa }$ remains confined within the interval $\left[1,\underline{\rho }\right]$.

The updating laws of $\hat{\Theta }$ and $\hat{\rho }$ are designed as follows:

(29) $$\begin{array}{cc} \dot{\hat{\Theta }}= & Proj_{\left[-\theta_{M},\;\theta_{M}\right]}\left\{2\gamma_{2}F\left({\hat{\kappa }}_{1}\chi_{1},{\hat{x}}_{n}\right)Pe_{10}-\sigma_{2}\hat{\Theta }\right\} \end{array}$$

(30) $$\begin{array}{cc} \dot{\hat{\rho }}= & Proj_{\left[\rho ,1\right]}\left(2\gamma_{0}e_{10}^{T}PB_{1}{\hat{x}}_{2}-\sigma_{0}\hat{\rho }\right) \end{array}$$

From the observer model, it is implied that κ(t)>0 for all t>0 if κ(0)≥0. In practice, it is reasonable to choose $\hat{\kappa }\left(0\right)\geq 0$, since $\hat{\kappa }$ is an estimate of κ. Since $\dot{\hat{\kappa }}\geq 0$ and $\tilde{\kappa }=\kappa -\hat{\kappa }$, and noting that $\lim_{t\to \infty }\hat{\kappa }\left(t\right)=\kappa$, it follows that $\hat{\kappa }\left(t\right)$ is monotonically non-decreasing and converges to κ. By substituting Equations (30) and (29) into Equation (26) and applying Young’s inequality, it can be obtained that

(31) $$\begin{array}{cc} V_{0} & \leq -e^{T}\bar{Q}\left(\kappa \chi_{1},\hat{x}\right)e+\frac{\sigma_{0}}{\gamma_{0}}\tilde{\rho }\dot{\hat{\rho }}+\frac{M\sigma_{1}}{\gamma_{1}}{\tilde{\kappa }}^{2}\dot{\hat{\kappa }}+\frac{\sigma_{2}}{\gamma_{2}}{\tilde{\Theta }}^{T}\dot{\hat{\Theta }}+{\tilde{\rho }}^{2}+{\tilde{\Theta }}^{T}\tilde{\Theta }+d_{M}^{2}\\ \; & \leq -e^{T}\bar{Q}\left(\kappa \chi_{1},\hat{x}\right)e-\left(\frac{\sigma_{0}}{2\gamma_{0}}-1\right){\tilde{\rho }}^{2}-\frac{M\sigma_{1}}{3\gamma_{1}}{\left|\tilde{\kappa }\right|}^{3}\\ \; & \;-\left(\frac{\sigma_{2}}{2\gamma_{2}}-1\right){\tilde{\Theta }}^{T}\tilde{\Theta }+\frac{\sigma_{0}}{2\gamma_{0}}\rho^{2}+\frac{M\sigma_{1}}{3\gamma_{1}}\kappa^{3}+\frac{\sigma_{2}}{2\gamma_{2}}\Theta^{T}\Theta +d_{M}^{2} \end{array}$$

From Equation (31), we can obtain the stability of the observer error system by choosing the design parameter $K=[k_{1},\ldots ,k_{n}]$ such that $\bar{Q}\left(\kappa x_{1},\hat{x}\right)>0$.

**Remark** **4.** *The stability of the system can be ensured via the designed piecewise update law $\dot{\hat{\kappa }}$. However, the available output signal may be attenuated due to the unknown output gain, which may degrade the tracking performance. To mitigate this effect, an additional modified term $\frac{\varepsilon \gamma_{1}^{\prime }}{\varepsilon \gamma_{1}^{\prime }+\chi_{1}^{2}}$ is incorporated into the update law in Equation (27), which enhances the compensation capability against output attenuation. Consequently, $\hat{\kappa }$ can increase rapidly when the output signal is affected by a small unknown gain.*

### 3.3. Design of Backstepping Adaptive Controller

An adaptive output-feedback control scheme with a state observer is constructed via the backstepping technique, ensuring the global boundedness of all signals in the closed-loop system.

First, the system is transformed into the following form:

(32) $$\begin{array}{cc} \; & {\dot{\chi }}_{1}=\rho x_{2}+\theta_{1}^{T}\phi_{1}\left(\kappa \chi_{1}\right)+\rho d_{1}\left(t\right)\\ \; & {\dot{\hat{x}}}_{i}={\hat{x}}_{i+1}+{\hat{\theta }}_{i}^{T}\phi_{i}\left({\hat{\kappa }}_{1}\chi_{1},{\bar{\hat{x}}}_{i}\right)+k_{i}\left(y^{F}-{\hat{x}}_{1}\right)\\ \; & {\dot{\hat{x}}}_{n}=u+{\hat{\theta }}_{n}^{T}\phi_{n}\left({\hat{\kappa }}_{1}\chi_{1},{\bar{\hat{x}}}_{n}\right)+k_{n}\left(y^{F}-{\hat{x}}_{1}\right) \end{array}$$

The controller design follows a backstepping procedure consisting of *n* steps. Different from conventional stability problems addressed by backstepping methods, a coordinate transformation is constructed as follows:

(33) $$\begin{array}{cc} \; & z_{1}=\hat{\kappa }\chi_{1}-y_{d}\\ \; & z_{i}={\hat{x}}_{i}-\alpha_{i-1},\;i=2,\ldots ,n \end{array}$$

We design the following universal transformed functions:

(34) $$\begin{array}{cc} \; & \varphi_{1}=\frac{z_{1}}{\lambda_{1}}\\ \; & \varphi_{i}=\frac{z_{i}}{\lambda_{i}},\;i=2,\ldots ,n \end{array}$$

Step 1:

According to the definition and properties of $\varphi_{1}$, we have

(35) $${\dot{\varphi }}_{1}=\mu_{1}\left[\dot{\hat{\kappa }}\chi_{1}+\hat{\kappa }\left(\rho x_{2}+\theta_{1}^{T}\phi_{1}\left(\kappa \chi_{1}\right)+\rho d_{1}\left(t\right)\right)-{\dot{y}}_{d}\right]+\zeta_{1}$$

We then construct the Lyapunov candidate function $V_{1}$:

(36) $$V_{1}=\frac{1}{2}\varphi_{1}^{2}+\frac{1}{2\gamma_{3}}{\tilde{\delta }}_{1}^{2}+\frac{1}{2\gamma_{4}}{\tilde{s}}^{2}$$

where ${\tilde{\delta }}_{1}=\delta_{1}-{\hat{\delta }}_{1}$ and $\tilde{s}=s-\hat{s}$.

According to the first equation of Equation (35), ${\dot{V}}_{1}$ can be obtained as follows:

(37) $$\begin{array}{cc} {\dot{V}}_{1}= & \varphi_{1}\mu_{1}\hat{\kappa }\rho \left({\tilde{x}}_{2}+z_{2}\right)+\varphi_{1}\mu_{1}\hat{\kappa }\rho \alpha_{1}+\varphi_{1}\mu_{1}\left[\hat{\kappa }\theta_{1}^{T}\phi_{1}\left(\kappa \chi_{1}\right)+\hat{\kappa }d_{1}\left(t\right)-{\dot{y}}_{d}\right]\\ \; & +\varphi_{1}\zeta_{1}+\varphi_{1}\mu_{1}\dot{\hat{\kappa }}\chi_{1}-\frac{1}{\gamma_{3}}{\tilde{\delta }}_{1}{\dot{\hat{\delta }}}_{1}-\frac{1}{\gamma_{4}}\tilde{s}\dot{\hat{s}} \end{array}$$

Since $\phi_{1j}\left(0\right)=0$, and $\phi_{1j}$ is smooth, there exists a continuous function ${\bar{\phi }}_{1j}$ such that $\phi_{1j}\left(\kappa \chi_{1}\right)=\kappa \chi_{1}{\bar{\phi }}_{1j}\left(\kappa x_{1}\right)$. On the other hand, according to Lemma 1, as for $\phi_{1j}\left(\kappa \chi_{1}\right)$, there exist $A_{j}\left(\cdot \right)$ and B(·) such that $|{\bar{\phi }}_{1j}\left(\kappa x_{1}\right)|\leq A_{j}\left(\kappa \right)B_{j}\left(\chi_{1}\right)$. We define $B\left(\chi_{1}\right)={\left[B_{1}\left(\chi_{1}\right),\ldots ,B_{p}\left(\chi_{1}\right)\right]}^{T}$, $\vartheta_{j}≜\kappa A_{j}\left(\kappa \right)\left|\theta_{1j}\right|$, and $\vartheta ={\left[\vartheta_{1},\ldots ,\vartheta_{p}\right]}^{T}$. Therefore, we have

(38) $$\begin{array}{c} \hat{\kappa }\theta_{1}^{T}\phi_{1}\left(\kappa \chi_{1}\right)\leq \hat{\kappa }\chi_{1}\vartheta B \end{array}$$

Then, Equation (37) becomes

(39) $$\begin{array}{cc} {\dot{V}}_{1}= & \varphi_{1}\mu_{1}\hat{\kappa }\rho \left({\tilde{x}}_{2}+z_{2}\right)+\varphi_{1}\mu_{1}\hat{\kappa }\rho \alpha_{1}+\varphi_{1}\mu_{1}\left[\hat{\kappa }\chi_{1}\vartheta B+\hat{\kappa }d_{1}\left(t\right)-{\dot{y}}_{d}\right]\\ \; & +\varphi_{1}\zeta_{1}+\varphi_{1}\mu_{1}\dot{\hat{\kappa }}\chi_{1}-\frac{1}{\gamma_{3}}{\tilde{\delta }}_{1}{\dot{\hat{\delta }}}_{1}-\frac{1}{\gamma_{4}}\tilde{s}\dot{\hat{s}} \end{array}$$

To address the bounded time-varying uncertainties, we define that $\Phi_{1}=\sup_{t\geq 0}∥\Xi_{1}\left(t\right)∥$ and

(40) $$\begin{array}{cc} \; & \Xi_{1}\left(t\right)={\left[\begin{array}{c} \vartheta ,\;d_{1}\left(t\right)-\frac{1}{\hat{\kappa }}{\dot{y}}_{d} \end{array}\right]}^{T}\in R^{p_{1}+1} \end{array}$$

(41) $$\begin{array}{cc} \; & \xi_{1}={\left[\begin{array}{c} \chi_{1}B,\;1 \end{array}\right]}^{T}\in R^{p_{1}+1} \end{array}$$

(42) $$\begin{array}{cc} \; & \delta_{1}=\max\left\{\mu_{d}^{M},\;\Phi_{1}\right\} \end{array}$$

Hence, we can find that

(43) $$\begin{array}{cc} \varphi_{1}\mu_{1}\hat{\kappa }\left[\theta_{1}^{T}\phi_{1}\left(\kappa \chi_{1}\right)+d_{1}\left(t\right)-{\dot{y}}_{d}\right] & =\varphi_{1}\mu_{1}\hat{\kappa }\left[\Xi_{1}^{T}\left(t\right)\xi_{1}\right]\\ \; & \leq |\varphi_{1}|\mu_{1}\hat{\kappa }\vartheta_{1}\left|\right|\xi_{1}\left|\right|\\ \; & \leq \delta_{1}\eta_{0}+\delta_{1}\varphi_{1}\mu \hat{\kappa }\tau \end{array}$$

where $\tau =(\frac{\varphi_{1}\mu \hat{\kappa }\xi_{1}^{T}\xi_{1}}{\sqrt{\varphi_{1}^{2}\mu^{2}{\hat{\kappa }}^{2}\xi_{1}^{T}\xi_{1}+\eta_{0}^{2}}})$.

With Young’s inequality, we also obtain that

(44) $$\begin{array}{c} \varphi_{1}\mu_{1}\hat{\kappa }\rho \left({\tilde{x}}_{2}+z_{2}\right)\leq \left(\frac{1}{2}+\frac{1}{\eta_{1}}\right)\varphi_{1}^{2}\mu_{1}^{2}{\hat{\kappa }}^{2}+\frac{\eta_{1}}{4}{\tilde{x}}_{2}^{2}+\frac{1}{2}\lambda_{2}^{2}\varphi_{2}^{2} \end{array}$$

Substituting Equations (43) and (44) into Equation (39) yields that

(45) $$\begin{array}{cc} {\dot{V}}_{1}\leq & \left(\frac{1}{2}+\frac{1}{\eta_{1}}\right)\varphi_{1}^{2}\mu_{1}^{2}{\hat{\kappa }}^{2}+\varphi_{1}\zeta_{1}+\varphi_{1}\mu_{1}\dot{\hat{\kappa }}\chi_{1}+\delta_{1}\varphi_{1}\mu_{1}\hat{\kappa }\tau +\varphi_{1}\mu_{1}\hat{\kappa }\rho \alpha_{1}\\ \; & +\frac{1}{2}\lambda_{2}^{2}\varphi_{2}^{2}+\delta_{1}\eta_{0}+\frac{\eta_{1}}{4}{\tilde{x}}_{2}^{2}-\frac{1}{\gamma_{3}}{\tilde{\delta }}_{1}{\dot{\hat{\delta }}}_{1}-\frac{\rho }{\gamma_{4}}\tilde{s}\dot{\hat{s}} \end{array}$$

We design the following virtual controller $\alpha_{1}=-\frac{\hat{s}}{\hat{\kappa }}{\bar{\alpha }}_{1}\frac{1}{\mu_{1}}$ with

(46) $$\begin{array}{cc} \; & {\bar{\alpha }}_{1}=c_{1}\varphi_{1}+\left(\frac{1}{2}+\frac{1}{\eta_{1}}\right)\varphi_{1}\mu_{1}^{2}{\hat{\kappa }}^{2}+\zeta_{1}+\mu_{1}\dot{\hat{\kappa }}\chi_{1}+{\hat{\delta }}_{1}\mu_{1}\hat{\kappa }\tau \end{array}$$

(47) $$\begin{array}{cc} \; & {\dot{\hat{\delta }}}_{1}=\gamma_{3}\varphi_{1}\mu_{1}\hat{\kappa }\tau -\gamma_{3}\sigma_{3}{\hat{\delta }}_{1} \end{array}$$

(48) $$\begin{array}{cc} \; & \dot{\hat{s}}=\gamma_{4}\varphi_{1}{\bar{\alpha }}_{1}-\gamma_{4}\sigma_{4}\hat{s} \end{array}$$

Then, it can be derived that

(49) $$\begin{array}{cc} \varphi_{1}\mu_{1}\hat{\kappa }\rho \alpha_{1} & \leq \varphi_{1}\mu_{1}\hat{\kappa }\rho \left(-\frac{\hat{s}}{\hat{\kappa }}{\bar{\alpha }}_{1}\frac{1}{\mu_{1}}\right)\\ \; & \leq -\varphi_{1}\rho \left(s-\tilde{s}\right){\bar{\alpha }}_{1}\leq -\varphi_{1}{\bar{\alpha }}_{1}+\varphi_{1}\rho \tilde{s}{\bar{\alpha }}_{1} \end{array}$$

Thus, we have

(50) $$\begin{array}{cc} {\dot{V}}_{1} & \leq -c_{1}\varphi_{1}^{2}+\frac{1}{2}\lambda_{2}^{2}\varphi_{2}^{2}+\delta_{1}\eta_{0}+\frac{\eta_{1}}{4}{\tilde{x}}_{2}^{2}-\frac{1}{\gamma_{3}}{\tilde{\delta }}_{1}\left({\dot{\hat{\delta }}}_{1}-\gamma_{3}\varphi_{1}\mu_{1}\hat{\kappa }\tau \right)-\frac{1}{\gamma_{4}}\tilde{s}\left(\dot{\hat{s}}-\gamma_{4}\varphi_{1}\rho {\bar{\alpha }}_{1}\right)\\ & \leq -c_{1}\varphi_{1}^{2}+\frac{1}{2}\lambda_{2}^{2}\varphi_{2}^{2}+\delta_{1}\eta_{0}+\frac{\eta_{1}}{4}{\tilde{x}}_{2}^{2}+\sigma_{3}{\tilde{\delta }}_{1}{\hat{\delta }}_{1}+\sigma_{4}\tilde{s}\hat{s} \end{array}$$

Step 2:

According to Equation (34), the derivative of $\phi_{i}$ can be expressed as follows:

(51) $$\begin{array}{cc} {\dot{\varphi }}_{2} & =\mu_{2}\left[{\dot{\hat{x}}}_{2}-{\dot{\alpha }}_{1}\right]+\zeta_{2} \end{array}$$

and the derivative of $z_{2}$ can be obtained:

(52) $$\begin{array}{cc} {\dot{z}}_{2}= & {\hat{x}}_{3}+{\hat{\theta }}_{2}^{T}\phi_{2}\left({\hat{\kappa }}_{1}\chi_{1},{\bar{\hat{x}}}_{2}\right)+k_{2}e_{1}-\frac{\partial \alpha_{1}}{\partial \chi_{1}}\left[\rho x_{2}+\theta_{1}^{T}\phi_{1}\left(\kappa \chi_{1}\right)+\rho d_{1}\left(t\right)\right]\\ \; & -\frac{\partial \alpha_{1}}{\partial {\hat{x}}_{j}}{\dot{\hat{x}}}_{j}-\frac{\partial \alpha_{1}}{\partial {\hat{\theta }}_{j}}{\dot{\hat{\theta }}}_{1}-\frac{\partial \alpha_{1}}{\partial {\hat{\kappa }}_{j}}{\dot{\hat{\kappa }}}_{1}-\frac{\partial \alpha_{1}}{\partial {\underline{H}}_{1}}{\dot{\underline{H}}}_{1}-\frac{\partial \alpha_{1}}{\partial {\bar{H}}_{1}}{\dot{\bar{H}}}_{1}\\ \; & -\frac{\partial \alpha_{1}}{\partial {\hat{\delta }}_{j}}{\dot{\hat{\delta }}}_{1}-\frac{\partial \alpha_{1}}{\partial {\hat{s}}_{j}}{\dot{\hat{s}}}_{1}-\frac{\partial \alpha_{1}}{\partial {\hat{y}}_{dj}}{\dot{\hat{y}}}_{d}-\frac{\partial \alpha_{1}}{\partial {\dot{\hat{y}}}_{dj}}{\ddot{\hat{y}}}_{d}\\ = & {\hat{x}}_{3}-\frac{\partial \alpha_{1}}{\partial \chi_{1}}\left[\rho x_{2}+\theta_{1}^{T}\phi_{1}\left(\kappa \chi_{1}\right)+\rho d_{1}\left(t\right)\right]+\Lambda_{2} \end{array}$$

Using Young’s inequality, it follows that

(53) $$\begin{array}{cc} \; & -\varphi_{2}\mu_{2}\frac{\partial \alpha_{1}}{\partial \chi_{1}}\rho x_{2}\leq \varphi_{2}^{2}\mu_{2}^{2}{\left(\frac{\partial \alpha_{1}}{\partial \chi_{1}}\right)}^{2}{\hat{x}}_{2}^{2}+\frac{1}{4}+\frac{1}{\eta_{1}}\varphi_{2}^{2}\mu_{2}^{2}{\left(\frac{\partial \alpha_{1}}{\partial \chi_{1}}\right)}^{2}+\frac{\eta_{1}}{4}e_{2}^{2} \end{array}$$

(54) $$\begin{array}{cc} \; & -\varphi_{2}\mu_{2}\frac{\partial \alpha_{1}}{\partial \chi_{1}}\theta_{1}^{T}\phi_{1}\left(\kappa \chi_{1}\right)\leq \varphi_{2}^{2}\mu_{2}^{2}{\left(\frac{\partial \alpha_{1}}{\partial \chi_{1}}\right)}^{2}\phi_{1}^{T}\left(\kappa \chi_{1}\right)\phi_{1}\left(\kappa \chi_{1}\right)+\frac{1}{4}\theta_{M}^{T}\theta_{M} \end{array}$$

(55) $$\begin{array}{cc} \; & -\varphi_{2}\mu_{2}\frac{\partial \alpha_{1}}{\partial \chi_{1}}\rho d_{1}\left(t\right)\leq \varphi_{2}^{2}\mu_{2}^{2}{\left(\frac{\partial \alpha_{1}}{\partial \chi_{1}}\right)}^{2}+\frac{1}{4}D^{2} \end{array}$$

We establish the Lyapunov function candidate:

(56) $$\begin{array}{c} V_{2}=\frac{1}{2}\varphi_{2}^{2} \end{array}$$

The time derivative of $V_{2}$ is

(57) $$\begin{array}{cc} {\dot{V}}_{2} & =\varphi_{2}\mu_{2}\left\{\lambda_{3}\varphi_{3}+\alpha_{2}-\frac{\partial \alpha_{1}}{\partial \chi_{1}}\left[\rho x_{2}+\theta_{1}^{T}\phi_{1}\left(\kappa \chi_{1}\right)+\rho d_{1}\left(t\right)\right]+\Lambda_{2}\right\}+\varphi_{2}\zeta_{2}\\ & \leq \varphi_{2}\mu_{2}\lambda_{3}\varphi_{3}+\varphi_{2}\mu_{2}\alpha_{2}+\varphi_{2}\zeta_{2}+\varphi_{2}^{2}\mu_{2}^{2}{\left(\frac{\partial \alpha_{1}}{\partial \chi_{1}}\right)}^{2}+\frac{1}{4}D^{2}+\varphi_{2}\mu_{2}\Lambda_{2}\\ & \;+\varphi_{2}^{2}\mu_{2}^{2}{\hat{x}}_{2}^{2}{\left(\frac{\partial \alpha_{1}}{\partial \chi_{1}}\right)}^{2}+\frac{1}{4}+\frac{1}{\eta_{1}}\varphi_{2}^{2}\mu_{2}^{2}{\left(\frac{\partial \alpha_{1}}{\partial \chi_{1}}\right)}^{2}+\frac{\eta_{1}}{4}e_{2}^{2}\\ & \;+\varphi_{2}^{2}\mu_{2}^{2}{\left(\frac{\partial \alpha_{1}}{\partial \chi_{1}}\right)}^{2}\phi_{1}^{T}\left(\kappa \chi_{1}\right)\phi_{1}\left(\kappa \chi_{1}\right)+\frac{1}{4}\theta_{M}^{T}\theta_{M} \end{array}$$

We design the virtual control $\alpha_{2}$ as follows:

(58) $$\alpha_{2}=-c_{i}\frac{\varphi_{2}}{\mu_{2}}-\frac{\varphi_{2}}{2\mu_{2}}\lambda_{2}^{2}-\frac{\zeta_{2}}{\mu_{2}}-\Lambda_{2}-\varphi_{2}\mu_{2}{\left(\frac{\partial \alpha_{1}}{\partial \chi_{1}}\right)}^{2}\left[1+{\hat{x}}_{2}^{2}+\frac{1}{\eta_{1}}+\phi_{1}^{T}\left(\kappa \chi_{1}\right)\phi_{1}\left(\kappa \chi_{1}\right)\right]$$

By substituting Equation (58) into Equation (57), we can obtain

(59) $${\dot{V}}_{2}\leq -c_{2}\varphi_{2}^{2}-\frac{1}{2}\lambda_{2}^{2}\varphi_{2}^{2}+\frac{1}{4}+\frac{\eta_{1}}{4}e_{2}^{2}+\frac{1}{4}\theta_{M}^{T}\theta_{M}+\frac{1}{4}D^{2}+\mu_{2}\lambda_{3}\varphi_{2}\varphi_{3}$$

Step *i*:

According to Equation (12), the derivative of $\varphi_{i}$ can be expressed as follows:

(60) $$\begin{array}{c} {\dot{\varphi }}_{i}=\mu_{i}\left[{\dot{\hat{x}}}_{i}-{\dot{\alpha }}_{i-1}\right]+\zeta_{i} \end{array}$$

and the derivative of $z_{i}$ can be described as

(61) $$\begin{array}{cc} {\dot{z}}_{i}= & {\hat{x}}_{i+1}+{\hat{\theta }}_{i}^{T}\phi_{i}\left({\hat{\kappa }}_{1}\chi_{1},{\bar{\hat{x}}}_{i}\right)+k_{i}e_{1}-\frac{\partial \alpha_{1}}{\partial \chi_{1}}\left[\rho x_{2}+\theta_{1}^{T}\phi_{1}\left(\kappa \chi_{1}\right)+\rho d_{1}\left(t\right)\right]\\ \; & -\sum_{j=1}^{i-1}\frac{\partial \alpha_{i-1}}{\partial {\hat{x}}_{j}}{\dot{\hat{x}}}_{j}-\sum_{j=1}^{i-1}\frac{\partial \alpha_{i-1}}{\partial {\hat{\theta }}_{j}}{\dot{\hat{\theta }}}_{j}-\sum_{j=1}^{i-1}\frac{\partial \alpha_{i-1}}{\partial {\hat{\kappa }}_{j}}{\dot{\hat{\kappa }}}_{j}-\sum_{j=1}^{i-1}\frac{\partial \alpha_{i-1}}{\partial {\underline{H}}_{j}}{\dot{\underline{H}}}_{j}-\sum_{j=1}^{i-1}\frac{\partial \alpha_{i-1}}{\partial {\bar{H}}_{j}}{\dot{\bar{H}}}_{j}\\ \; & -\sum_{j=1}^{i-1}\frac{\partial \alpha_{i-1}}{\partial {\hat{\delta }}_{j}}{\dot{\hat{\delta }}}_{j}-\sum_{j=1}^{i-1}\frac{\partial \alpha_{i-1}}{\partial {\hat{s}}_{j}}{\dot{\hat{s}}}_{1}-\sum_{j=1}^{i-1}\frac{\partial \alpha_{i-1}}{\partial {\hat{y}}_{dj}}{\dot{\hat{y}}}_{dj}-\sum_{j=1}^{i-1}\frac{\partial \alpha_{i-1}}{\partial {\dot{\hat{y}}}_{dj}}{\ddot{\hat{y}}}_{dj}\\ = & {\hat{x}}_{i+1}-\frac{\partial \alpha_{i-1}}{\partial \chi_{1}}\left[\rho x_{2}+\theta_{1}^{T}\phi_{1}\left(\kappa \chi_{1}\right)+\rho d_{1}\left(t\right)\right]+\Lambda_{i} \end{array}$$

We establish the Lyapunov function candidate:

(62) $$\begin{array}{c} V_{i}=\frac{1}{2}\varphi_{i}^{2} \end{array}$$

and its derivative can be deduced as follows:

(63) $$\begin{array}{cc} {\dot{V}}_{i}= & \varphi_{i}\mu_{i}\left\{\lambda_{i+1}\varphi_{i+1}+\alpha_{i}-\frac{\partial \alpha_{i-1}}{\partial \chi_{1}}\left[\rho x_{2}+\theta_{1}^{T}\phi_{1}\left(\kappa \chi_{1}\right)+\rho d_{1}\left(t\right)\right]+\Lambda_{i}\right\}+\varphi_{i}\zeta_{i}\\ = & \varphi_{i}\mu_{i}\lambda_{i+1}\varphi_{i+1}+\varphi_{i}\mu_{i}\alpha_{i}+\varphi_{i}\mu_{i}\Lambda_{i}+\varphi_{i}\zeta_{i}\\ \; & -\varphi_{i}\mu_{i}\frac{\partial \alpha_{i-1}}{\partial \chi_{1}}\rho x_{2}-\varphi_{i}\mu_{i}\frac{\partial \alpha_{i-1}}{\partial \chi_{1}}\theta_{1}^{T}\phi_{1}\left(\kappa \chi_{1}\right)-\varphi_{i}\mu_{i}\frac{\partial \alpha_{i-1}}{\partial \chi_{1}}\rho d_{1}\left(t\right) \end{array}$$

By using Young’s inequality, it follows that

(64) $$-\varphi_{i}\mu_{i}\frac{\partial \alpha_{i-1}}{\partial \chi_{1}}\rho x_{2}\leq \varphi_{i}^{2}\mu_{i}^{2}{\left(\frac{\partial \alpha_{i-1}}{\partial \chi_{1}}\right)}^{2}{\hat{x}}_{2}^{2}+\frac{1}{4}+\frac{1}{\eta_{1}}\varphi_{i}^{2}\mu_{i}^{2}{\left(\frac{\partial \alpha_{i-1}}{\partial \chi_{1}}\right)}^{2}+\frac{\eta_{1}}{4}e_{2}^{2}$$

(65) $$-\varphi_{i}\mu_{i}\frac{\partial \alpha_{i-1}}{\partial \chi_{1}}\theta_{1}^{T}\phi_{1}\left(\kappa \chi_{1}\right)\leq \varphi_{i}^{2}\mu_{i}^{2}{\left(\frac{\partial \alpha_{i-1}}{\partial \chi_{1}}\right)}^{2}\phi_{1}^{T}\left(\kappa \chi_{1}\right)\phi_{1}\left(\kappa \chi_{1}\right)+\frac{1}{4}\theta_{M}^{T}\theta_{M}$$

(66) $$-\frac{\partial \alpha_{i-1}}{\partial \chi_{1}}\rho d_{1}\left(t\right)\leq \varphi_{i}^{2}\mu_{i}^{2}{\left(\frac{\partial \alpha_{i-1}}{\partial \chi_{1}}\right)}^{2}+\frac{1}{4}D^{2}$$

We design the virtual controller $\alpha_{i}$ as follows:

(67) $$\begin{array}{cc} \alpha_{i}= & -c_{i}\frac{1}{\mu_{i}}\varphi_{i}-\frac{\mu_{i-1}}{\mu_{i}}\lambda_{i}\varphi_{i-1}-\frac{\zeta_{i}}{\mu_{i}}-\varphi_{i}\mu_{i}{\left(\frac{\partial \alpha_{i-1}}{\partial \chi_{1}}\right)}^{2}[1+{\hat{x}}_{2}^{2}+\frac{1}{\eta_{1}}\\ \; & +\phi_{1}^{T}\left(\kappa \chi_{1}\right)\phi_{1}\left(\kappa \chi_{1}\right)]-\Lambda_{i} \end{array}$$

By combining this with Equation (67), we can obtain

(68) $${\dot{V}}_{i}\leq -c_{i}\varphi_{i}^{2}+\frac{1}{4}+\frac{\eta_{1}}{4}e_{2}^{2}+\frac{1}{4}\theta_{M}^{T}\theta_{M}+\frac{1}{4}D^{2}+\mu_{i}\lambda_{i+1}\varphi_{i}\varphi_{i+1}-\mu_{i-1}\lambda_{i}\varphi_{i-1}\varphi_{i}$$

Step *n*:

We can get that ${\dot{z}}_{n}=u+{\hat{\theta }}_{n}^{T}\phi_{n}\left({\hat{\kappa }}_{1}\chi_{1},{\bar{\hat{x}}}_{n}\right)+k_{n}\left(y^{F}-{\hat{x}}_{1}\right)$ and

(69) $$\begin{array}{c} {\dot{z}}_{n}=u-\frac{\partial \alpha_{n-1}}{\partial \chi_{1}}\left[\rho x_{2}+\theta_{1}^{T}\phi_{1}\left(\kappa \chi_{1}\right)+\rho d_{1}\left(t\right)\right]+\Lambda_{n} \end{array}$$

with

(70) $$\begin{array}{cc} \Lambda_{n}= & {\hat{\theta }}_{n}^{T}\phi_{n}\left({\hat{\kappa }}_{1}\chi_{1},{\bar{\hat{x}}}_{n}\right)+k_{n}e_{1}-\sum_{j=1}^{n-1}\frac{\partial \alpha_{n-1}}{\partial {\hat{x}}_{j}}{\dot{\hat{x}}}_{j}-\sum_{j=1}^{n-1}\frac{\partial \alpha_{n-1}}{\partial {\hat{\theta }}_{j}}{\dot{\hat{\theta }}}_{j}\\ \; & -\sum_{j=1}^{n-1}\frac{\partial \alpha_{n-1}}{\partial {\hat{\kappa }}_{j}}{\dot{\hat{\kappa }}}_{j}-\sum_{j=1}^{n-1}\frac{\partial \alpha_{n-1}}{\partial {\underline{H}}_{j}}{\dot{\underline{H}}}_{j}-\sum_{j=1}^{n-1}\frac{\partial \alpha_{n-1}}{\partial {\bar{H}}_{j}}{\dot{\bar{H}}}_{j}\\ \; & -\sum_{j=1}^{n-1}\frac{\partial \alpha_{n-1}}{\partial {\hat{\delta }}_{j}}{\dot{\hat{\delta }}}_{j}-\sum_{j=1}^{n-1}\frac{\partial \alpha_{n-1}}{\partial {\hat{s}}_{j}}{\dot{\hat{s}}}_{1}-\sum_{j=1}^{n-1}\frac{\partial \alpha_{n-1}}{\partial {\hat{y}}_{dj}}{\dot{\hat{y}}}_{dj}-\sum_{j=1}^{n-1}\frac{\partial \alpha_{n-1}}{\partial {\dot{\hat{y}}}_{dj}}{\ddot{\hat{y}}}_{dj} \end{array}$$

We construct the Lyapunov function candidate:

(71) $$\begin{array}{c} V_{n}=\frac{1}{2}\varphi_{n}^{2} \end{array}$$

The time derivative of $V_{n}$ is

(72) $$\begin{array}{cc} {\dot{V}}_{n}= & \varphi_{n}\mu_{n}\left\{u-\frac{\partial \alpha_{n-1}}{\partial \chi_{1}}\left[\rho x_{2}+\theta_{1}^{T}\phi_{1}\left(\kappa \chi_{1}\right)+\rho d_{1}\left(t\right)\right]+\Lambda_{n}\right\}+\varphi_{n}\zeta_{n} \end{array}$$

We design the following adaptive controller:

(73) $$\begin{array}{cc} u= & -c_{n}\frac{1}{\mu_{n}}\varphi_{n}-\frac{\mu_{n-1}}{\mu_{n}}\lambda_{n}\varphi_{n-1}-\frac{\zeta_{n}}{\mu_{n}}-\Lambda_{n}\\ \; & -\varphi_{n}\mu_{n}{\left(\frac{\partial \alpha_{n-1}}{\partial \chi_{1}}\right)}^{2}\left[1+{\hat{x}}_{2}^{2}+\frac{1}{\eta_{1}}+\phi_{1}^{T}\left(\kappa \chi_{1}\right)\phi_{1}\left(\kappa \chi_{1}\right)\right] \end{array}$$

and hence, it follows that

(74) $${\dot{V}}_{n}=-c_{n}\varphi_{n}^{2}+\frac{1}{4}+\frac{\eta_{1}}{4}e_{2}^{2}+\frac{1}{4}\theta_{M}^{T}\theta_{M}+\frac{1}{4}D^{2}-\mu_{n-1}\lambda_{n}\varphi_{n-1}\varphi_{n}$$

### 3.4. Stability Analysis

The main results of the proposed method are formally summarized in the following theorem at this stage.

**Theorem** **1.** *Under Assumptions 1–4, considering the parametric strict feedback systems subject to unknown output gain and asymmetric time-varying full-state constraints, with the designed adaptive state observer in Equation* (16)*, the error transformations in Equations* (7) *and* (12)*, the adaptive updating laws in Equations* (29) *and* (30)*, and the adaptive tracking controller in Equation* (73)*, the following results can be obtained:*

*(1)*  *All signals in the closed-loop system remain uniformly bounded;*
*(2)*  *The system states always evolve within the time-varying asymmetric constraint bounds.*

**Proof.**  Selecting the Lypunov function $V=V_{0}+V_{1}+\ldots +V_{n}$ then yields that

(75) $$\begin{array}{cc} {\dot{V}}_{all}\leq & -e^{T}\left(\bar{Q}-\frac{n\eta_{1}}{2}I_{2}\right)e-\left(c_{1}-\frac{1}{2}\right)\varphi_{1}^{2}-\sum_{i=2}^{n}c_{i}\varphi_{i}^{2}-\left(\frac{\sigma_{0}}{2\gamma_{0}}-1\right){\tilde{\rho }}^{2}\\ \; & -\frac{M\sigma_{1}}{3\gamma_{1}}{\left|\tilde{\kappa }\right|}^{3}-\left(\frac{\sigma_{2}}{2\gamma_{2}}-1\right){\tilde{\Theta }}^{T}\tilde{\Theta }+\frac{n-1}{2}\theta_{M}^{T}\theta_{M}-\left(1-\frac{1}{\zeta_{3}}\right)\sigma_{3}{\tilde{\delta }}_{1}^{2}\\ \; & +a_{0}\sqrt{\eta }+0.2785\;mD+0.2785\;m\Xi +\zeta_{3}\sigma_{3}\delta_{1}^{2}+\zeta_{4}\sigma_{4}s^{2}\\ \; & +d_{M}^{2}+\frac{\sigma_{0}}{2\gamma_{0}}\rho^{2}+\frac{M\sigma_{1}}{3\gamma_{1}}\kappa^{3}+\frac{\sigma_{2}}{2\gamma_{2}}\Theta^{T}\Theta -\left(1-\frac{1}{\zeta_{4}}\right)\sigma_{4}{\tilde{s}}^{2}\\ \leq & -\Lambda V+\iota \end{array}$$

where $I_{2}=diag\left\{0,1,\ldots ,0\right\}$, $\Lambda =\min\left\{\frac{\lambda_{\min}\;\left[\bar{Q}-\frac{n\eta_{1}}{2}I_{2}\right]}{\lambda_{\max}\left(P\right)},\frac{2M\sigma_{1}}{3\gamma_{1}},2\left(\frac{\sigma_{0}}{2\gamma_{0}}-1\right),2\left(\frac{\sigma_{2}}{2\gamma_{2}}-1\right),2\left(c_{1}-\frac{1}{2}\right),2c_{2},\ldots ,2c_{n},2\sigma_{3}\left(1-\frac{1}{\zeta_{3}}\right),2\sigma_{4}\left(1-\frac{1}{\zeta_{4}}\right)\right\}$, and $\iota =d_{M}^{2}+\frac{\sigma_{0}}{2\gamma_{0}}\rho^{2}+\frac{M\sigma_{1}}{3\gamma_{1}}\kappa^{3}+\frac{\sigma_{2}}{2\gamma_{2}}\Theta^{T}\Theta +\frac{n-1}{2}\theta_{M}^{T}\theta_{M}+0.2785m\Xi +0.2785mD+a_{0}\sqrt{\eta }+\zeta_{3}\sigma_{3}\delta_{1}^{2}+\zeta_{4}\sigma_{4}s^{2}$. □

To guarantee the stability of the closed-loop systems, the parameters $c_{1},\ldots ,c_{n}$, $\sigma_{0}$, $\sigma_{1}$, $\sigma_{2}$, $\zeta_{3}$, and $\zeta_{4}$ should be chosen to satisfy the following conditions: $c_{1}-\frac{1}{2}>0$, $c_{2},\ldots ,c_{n}>0$, $\frac{\sigma_{0}}{2\gamma_{0}}-1>0$, $\frac{\sigma_{2}}{2\gamma_{2}}-1>0$, $1-\frac{1}{\zeta_{3}}>0$, and $1-\frac{1}{\zeta_{4}}>0$.

To ensure the stability of the closed-loop system, the observer gain parameter *k* is designed such that the following matrix inequality is satisfied:

(76) $$A^{T}P+PA-2PP-\Psi \left(t\right){\bar{I}}_{1}PP{\bar{I}}_{1}+\beta^{2}PB_{n}B_{n}^{T}P-\theta_{M}^{2}\sum_{i=1}^{n}\left(L_{i}^{2}\right)+\frac{n\eta_{1}}{4}<0$$

where $\Psi \left(t\right)=\max_{1\leq i\leq n}\phi_{i}\left({\hat{x}}_{i}\right)$.

Based on the Schur complement, the aforementioned inequality is equivalent to the following condition:

(77) $$\begin{array}{c} \left[\begin{array}{cccc} \left(1,1\right) & * & * & *\\ {\bar{I}}_{1}P & -\Psi^{-1}I & * & *\\ P & 0 & -I & *\\ \beta B_{n}^{T}P & 0 & 0 & -I \end{array}\right]<0 \end{array}$$

To eliminate the bilinear terms involving *P* and *K*, a new variable C=KP is introduced. Accordingly, the controller gain can be recovered as $K=P^{-1}C$, which enables the conditions to be expressed in linear matrix inequality (LMI) form.

In the inequality, the (1,1) block is given by ${\bar{A}}^{T}P+P\bar{A}+H^{T}W+WH+\phi I$, $\bar{A}=\left[\begin{array}{ccc} 0 & & \\ \vdots & \hat{G} & \\ 0 & \cdots & 0 \end{array}\right]$, $H=[\begin{array}{ccccc} -1 & 0 & \cdots & 0 & 0 \end{array}]$. and $K=P^{-1}C$. Moreover, $\hat{G}\in \left\{G_{1},G_{2}\right\}$ with $G_{1}=\mathrm{diag}\left\{1,1,\ldots ,1\right\}$ and $G_{2}=I_{n-1}$.

It follows from integrating Equation (75) that

(78) $$V\left(t\right)\leq e^{-\Lambda t}V\left(0\right)+\frac{\iota }{\Lambda }(1-e^{-\Lambda t}).$$

Therefore, V(t) is bounded, and hence, all signals in the closed-loop systems remain bounded. That aside, the asymmetric time-varying constrains can be achieved.

**Remark** **5.** *From the stability result $\dot{V}\leq -\Lambda V+\iota$, the ultimate bound of the tracking error is determined by the constants* Λ *and ι. A larger* Λ *or a smaller ι leads to a tighter ultimate bound $\sqrt{2\iota /\Lambda }$. According to Equation (75), increasing the design parameters $c_{i},\sigma_{1},\ldots ,\sigma_{4}$ enlarges* Λ*, while increasing $\gamma_{0},\gamma_{1},\gamma_{2}$ helps reduce ι. However, excessively large $c_{i},\sigma_{1},\ldots ,\sigma_{4}$ values inherently induce high-gain control and increase the actuator burden. Thus, a tradeoff between performance and control effort should be adjusted by actual requirements.*

## 4. Simulation

To verify the effectiveness of the designed output-feedback adaptive tracking control scheme under unknown output gain, numerical simulations were carried out on a permanent magnet DC motor model. The dynamic model of the permanent magnet DC motor was adopted from [29,30] and, it is given by

(79) $$\left\{\begin{array}{c} {\dot{x}}_{1}=x_{2},\\ {\dot{x}}_{2}=-\frac{B}{J}x_{2}+\frac{K_{t}}{J}x_{3}-\frac{F_{c}}{J}\sin\left(x_{2}\right),\\ {\dot{x}}_{3}=\frac{1}{L_{q}}u-\frac{R_{a}}{L_{q}}x_{3}-\frac{1}{L_{q}}\left(1-\alpha x_{3}^{2}\right)x_{2}, \end{array}\right.$$

where the state variables are chosen as $x_{1}$, $x_{2}$, and $x_{3}$, representing the rotor angular position, angular velocity, and armature current, respectively. The control input *u* is defined as the applied armature voltage, while *y* denotes the measured system output. The parameter *J* denotes the rotor moment of inertia, *B* is the viscous damping factor, and $K_{t}$ represents the torque constant. $R_{s}$ denotes the armature resistance, and $L_{q}$ denotes the inductance.

The available output signal was assumed to be affected by unknown output gain. Specifically, the output gain model is given by

(80) $$y^{F}\left(t\right)=\left\{\begin{array}{cc} y(t), & t<4s,\\ 0.5y(t), & t\geq 4s, \end{array}\right.$$

The model in Equation (80) represents a typical multiplicative output gain attenuation. The activation time and attenuation level were selected as representative simulation settings to evaluate the tracking performance under unknown output gain.

The selected parameters of the DC motor system were $J=1\times 10^{-4}\;\mathrm{kg}\cdot m^{2}$, $B=1\times 10^{-4}\;N\cdot m\cdot s/\mathrm{rad}$, $R_{s}=2.0\;\Omega$, $L_{q}=5\times 10^{-3}\;H$, and $K_{t}=0.05\;N\cdot m/A$. The chosen desired reference trajectory was $y_{d}=0.2\sin\left(\pi t\right)$. The selected initial conditions of the model states were $x_{3}\left(0\right)=x_{2}\left(0\right)=0.2$, $x_{1}\left(0\right)=0.1$, ${\hat{x}}_{3}\left(0\right)={\hat{x}}_{2}\left(0\right)={\hat{x}}_{1}\left(0\right)=0.1$, $\hat{\rho }\left(0\right)=0.5$, $\hat{\kappa }\left(0\right)=1$, ${\hat{\delta }}_{1}\left(0\right)=0.1$, and $\hat{s}\left(0\right)=0.1$. To ensure safe operation of the system, time-varying state constraints were imposed on each state variable. The constraint boundaries were defined as ${\bar{H}}_{1}\left(t\right)=0.05\cos\left(t\right)+0.35$, ${\underline{H}}_{1}\left(t\right)=0.05\sin\left(t\right)-0.35$, ${\bar{H}}_{2}\left(t\right)=0.1\cos\left(t\right)+2.8$, ${\underline{H}}_{2}\left(t\right)=0.1\sin\left(t\right)-2.0$, ${\bar{H}}_{3}\left(t\right)=0.05\cos\left(t\right)+2.5$, and ${\underline{H}}_{3}\left(t\right)=0.1\sin\left(t\right)-3.0$. We chose the following design parameters: $c_{1}=120.5$, $c_{2}=1$, $c_{5}=5$, $\gamma_{0}=1055$, $\gamma_{1}=55$, $\gamma_{2}=1000$, $\gamma_{3}=1500$, $\gamma_{4}=1.1$, $\sigma_{0}=0.0005$, $\sigma_{1}=0.0001$, $\sigma_{2}=0.0005$, $\sigma_{3}=0.0005$, and $\sigma_{4}=0.0005$.

**Figure 1:**
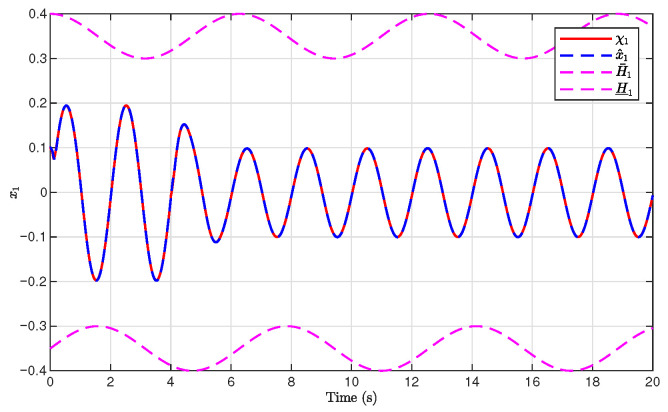
Evolution of $x_{1}$ and ${\hat{x}}_{1}$ with constraint boundaries ${\bar{H}}_{1}$ and ${\underline{H}}_{1}$.

**Figure 2:**
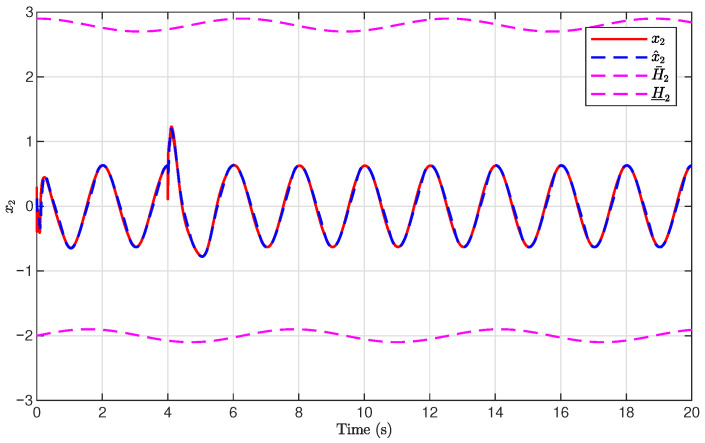
Evolution of $x_{2}$ and ${\hat{x}}_{2}$ with constraint boundaries ${\bar{H}}_{2}$ and ${\underline{H}}_{2}$.

**Figure 3:**
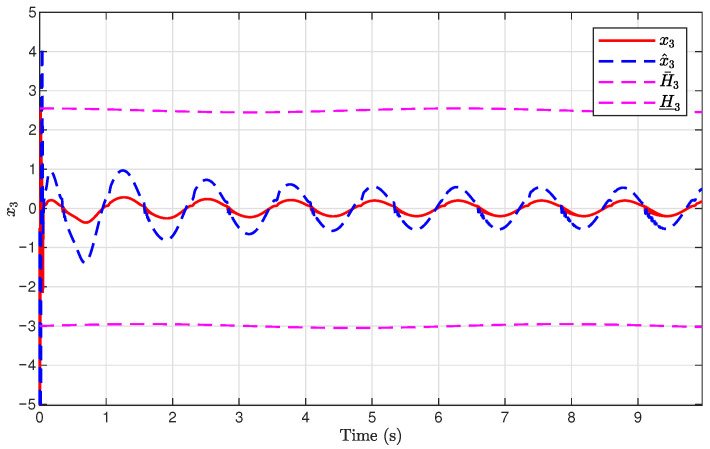
Evolution of $x_{3}$ and ${\hat{x}}_{3}$ with constraint boundaries ${\bar{H}}_{3}$ and ${\underline{H}}_{3}$.

**Figure 4:**
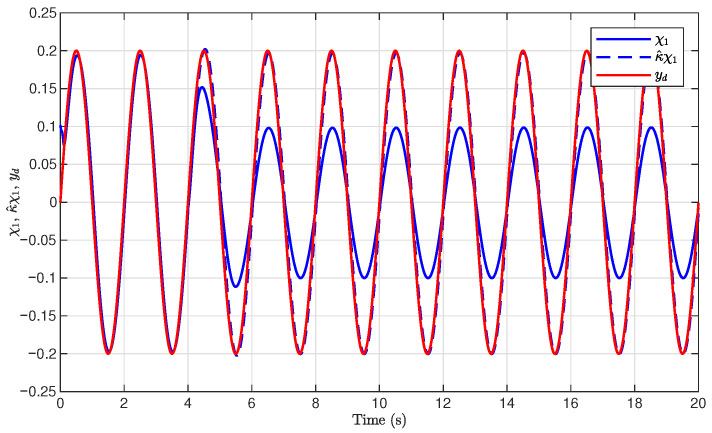
Tracking performance of the output $x_{1}$, $\chi_{1}$, and $y_{d}$.

**Figure 5:**
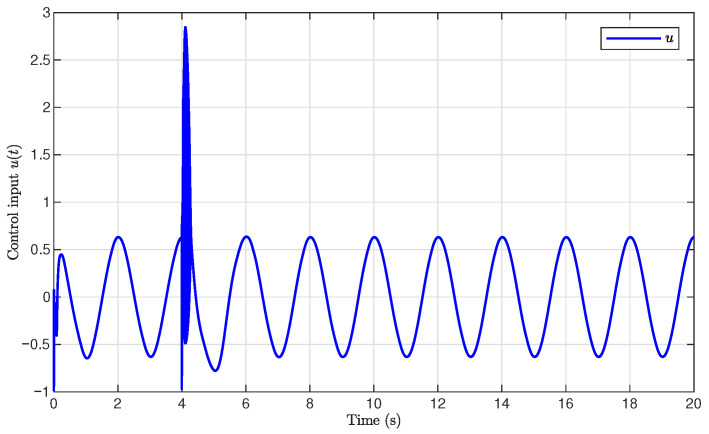
Control input signal u(t).

**Figure 6:**
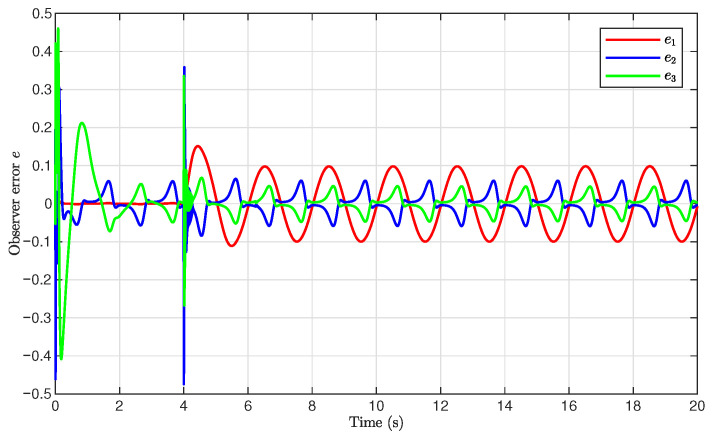
Observer errors $e_{1}$, $e_{2}$, and $e_{3}$.

**Figure 7:**
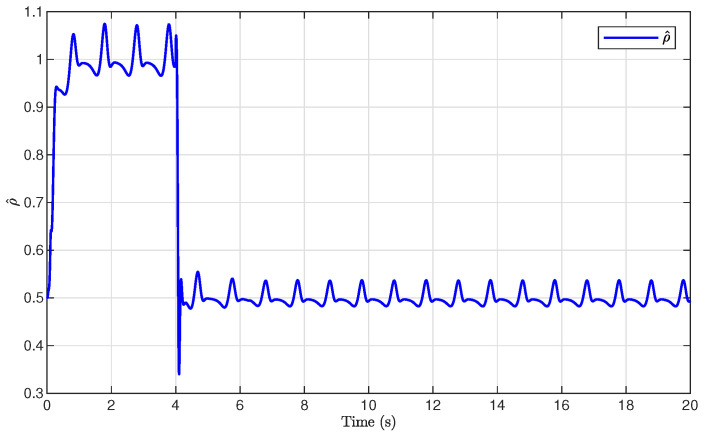
Evolution of the adaptive parameter $\hat{\rho }$.

**Figure 8:**
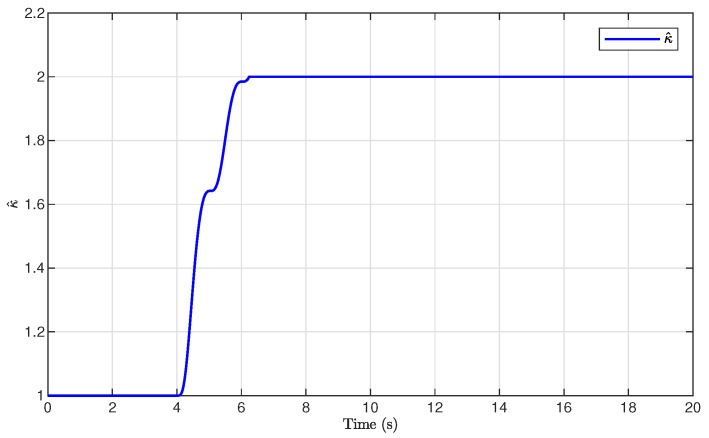
Evolution of the adaptive parameter $\hat{\kappa }$.

**Figure 9:**
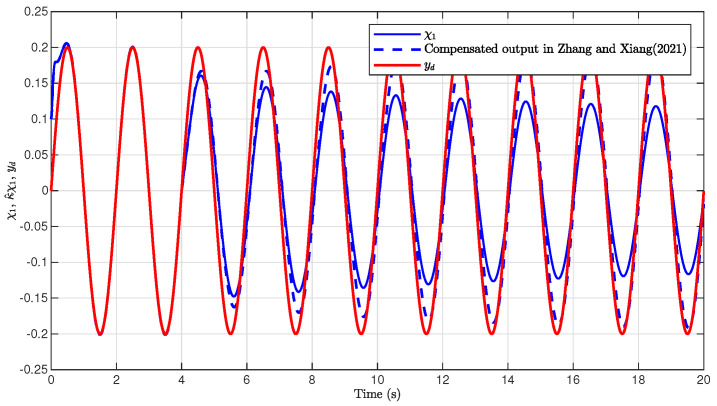
Tracking performance of the output $x_{1}$, $\chi_{1}$, and $y_{d}$ in [17].

The simulation results are presented in Figure 1, Figure 2, Figure 3, Figure 4, Figure 5, Figure 6, Figure 7, Figure 8 and Figure 9. Figure 1, Figure 2 and Figure 3 illustrate the evolutions of the system states $x_{1}$, $x_{2}$, and $x_{3}$ together with their estimates ${\hat{x}}_{1}$, ${\hat{x}}_{2}$, and ${\hat{x}}_{3}$, as well as the constraint boundaries ${\bar{H}}_{1}$, ${\underline{H}}_{1}$, ${\bar{H}}_{2}$, ${\underline{H}}_{2}$, ${\bar{H}}_{3}$, and ${\underline{H}}_{3}$, respectively. It can be observed that the estimated states closely tracked the actual system states in the presence of unknown output gain. Moreover, all state trajectories remained strictly within the asymmetric constraint boundaries. These results demonstrate that the proposed adaptive output-feedback controller can effectively guarantee state constraint satisfaction while maintaining accurate state estimation under unknown output gain.

Figure 4 illustrates the tracking performance of the proposed method and its comparison with the method in [17]. As shown in Figure 4, the system output $x_{1}$ can rapidly track the desired trajectory $y_{d}$ with high accuracy, even when the available output signal is affected by unknown output gain. The transient response was fast, and the steady-state tracking error remained small, demonstrating the effectiveness of the proposed adaptive compensation mechanism.

Figure 5, Figure 6, Figure 7 and Figure 8 illustrate the observer errors and the evolution of the adaptive parameters. As shown in Figure 6, the observer errors $e_{1}$, $e_{2}$, and $e_{3}$ converged to a small neighborhood around zero, indicating the effectiveness of the proposed state observer. Figure 7 presents the evolution of the adaptive parameter $\hat{\rho }$, which gradually converged with small oscillations during the transient process. Figure 8 shows the evolution of the adaptive compensation coefficient $\hat{\kappa }$. It can be observed that $\hat{\kappa }$ was adaptively adjusted to reduce the influence of unknown output gain, thereby maintaining accurate system output tracking.

For comparison, the compensation-based control method in [17] was applied. The simulation results are illustrated in Figure 9. It can be concluded from Figure 4 and Figure 9 that the proposed method achieved improved tracking performance compared with the method in [17]. In addition, the root mean square error (RMSE), the mean absolute error (MAE), and the integral absolute error (IAE) are presented in Table 1, demonstrating the advantages of the proposed adaptive compensation method in this paper.

## 5. Conclusions

This paper proposed an adaptive output-feedback tracking control strategy for nonlinear strict feedback systems subject to unknown output gain and full-state constraints. An adaptive state observer was constructed to estimate the unmeasured states under unknown output gain. Furthermore, by combining the UTFs with the bounded estimation method, the proposed control framework effectively addressed the state constraint problem. Based on the backstepping technique, a new error signal containing an adaptive compensation coefficient was introduced into the control design to reduce the influence of unknown output gain. It was shown that the closed-loop system was globally uniformly ultimately bounded, and the tracking error converged to an adjustable neighborhood of the origin. Simulation results confirmed the effectiveness and reliability of the proposed method. Future work will focus on extending the proposed framework to more general output gain uncertainties and actuator nonlinearities in nonlinear systems with state constraints. 

## Figures and Tables

**Table 1:** Performance comparison.

Performance Metrics	*ERMS*	*MAE*	*IAE*
Ours	0.012845	0.010867	0.217339
In [17]	0.016739	0.011610	0.232208

## Data Availability

The original contributions presented in this study are included in the article. Further inquiries can be directed to the corresponding authors.
